# The Diagnostic Value of Confocal Laser Endomicroscopy in Brain Tumours When Performed by Blinded, Untrained Neuropathologists

**DOI:** 10.1111/nan.70049

**Published:** 2026-01-16

**Authors:** Ilona Iff, Marielena Gutt‐Will, Theoni Maragkou, Andrea Mathis, Kathleen Seidel, David Capper, Elisabeth G. Hain, Jenny Meinhardt, Regina Von Manitius, Carsten Dittmayer, Simone Schmid, Ekkehard Hewer, Andreas Raabe, Philippe Schucht

**Affiliations:** ^1^ Department of Neurosurgery, Inselspital, Bern University Hospital and University of Bern Bern Switzerland; ^2^ Psychiatric Services Solothurn Solothurn Switzerland; ^3^ Institute of Tissue Medicine and Pathology University of Bern Bern Switzerland; ^4^ Department of General Internal Medicine Inselspital, Bern University Hospital, and University of Bern Switzerland; ^5^ Department of Neuropathology, Charité – Universitätsmedizin Berlin Berlin Germany; ^6^ German Cancer Consortium (DKTK) berlin Germany; ^7^ German Cancer Research Center (DKFZ) Heidelberg Germany; ^8^ Institute of Pathology, CHUV, University Hospital of Lausanne Lausanne Switzerland

**Keywords:** brain tumour, confocal laser endomicroscopy, glioma, meningioma, metastasis

## Abstract

**Aim:**

Achieving maximal and safe tumour resection is a key goal in brain tumour surgery. Confocal laser endomicroscopy (CLE) enables real‐time visualisation of the tissue microstructure at a cellular level, potentially helping neurosurgeons distinguish non‐neoplastic from neoplastic tissue. The core aim of this study was to determine the baseline diagnostic accuracy that can be achieved with CLE alone, when assessed by neuropathologists without prior CLE training and without any additional clinical or contextual information, and to compare these findings to standard haematoxylin and eosin (H & E)‐based histology in a blinded setting.

**Methods:**

CLE images and corresponding H & E‐stained slides from 100 brain tumour patients treated at the University Hospital Bern over a 22‐month period were analysed. Five blinded neuropathologists with no prior CLE experience independently evaluated the data sets.

**Results:**

Based on CLE images, neuropathologists differentiated neoplastic from non‐neoplastic tissue in 70.7%. The specific tumour type was correctly identified in 47.5%: gliomas in 59.8%, meningiomas in 43.8%, and metastases in 25.7%. In contrast, H & E slides were correctly classified as neoplastic in 87.6%, with 89.1% tumour‐type‐level accuracy (gliomas 85.6%, meningiomas 94.6%, metastases 88.2%). Confidence levels for CLE diagnoses were generally low, and no learning curve was observed.

**Conclusions:**

CLE shows potential to distinguish between neoplastic and non‐neoplastic tissue, but diagnostic accuracy remains lower than with H & E‐stained slides. Training in CLE image interpretation is recommended to improve diagnostic accuracy. CLE imaging may have the potential to become a valuable tool for delineating brain tumour borders.

## Summary

- Confocal Laser Endomicroscopy (CLE) enables visualisation of tissue microstructure on a cellular level
- CLE shows good potential to distinguish neoplastic from non‐neoplastic tissue
- To improve diagnostic accuracy, prior training in CLE image interpretation is recommended
- CLE could potentially become a tool to demarcate tumour borders

**Abbreviations**
CLE: confocal laser endomicroscopy
H & E: haematoxylin and eosin
FNa: sodium fluorescein
NaCl: sodium chloride
LGG: low‐grade glioma
HGG: high‐grade glioma
PPV: positive predictive value
NPV: negative predictive value
TP: true positive
TN: true negative
FP: false positive
FN: false negative

## 1 Introduction

Gliomas are the most prevalent form of malignant primary brain tumours and are broadly speaking categorised into high‐ and low‐grade variants. The classification of gliomas is based on histopathological features and molecular markers, both of which are linked to distinct prognoses [1, 2, 3]. Tumour tissue is obtained either through cytoreductive resection or biopsy. However, the diagnostic value of biopsies is considered to be limited as it has been associated with under‐grading [4].

The poor prognosis seen in glioma patients is partly due to the challenge of achieving a complete and safe tumour resection. This difficulty arises from the challenge in determining tumour margins, which is important as the overall survival of patients is closely linked to the extent of surgical resection [5, 6, 7, 8]. In addition, new postoperative neurological deficits are associated with lower overall survival [9]. Numerous novel techniques for brain tumour surgery have been investigated to improve the visualisation of tumour margins, potentially enabling a safer and more complete resection [10, 11].

Combining confocal laser endomicroscopy (CLE) with a contrast agent represents a promising innovation in this field. This technique allows visualisation of the tissue microstructure at a cellular level by applying a hand‐held laser directly onto the tissue during surgery and generating real‐time images [12, 13]. This technology has the potential to support surgeons in distinguishing non‐neoplastic from neoplastic tissue as well as to differentiate between tumour types [12, 13, 14, 15, 16, 17]. However, the accurate interpretation of CLE images remains the key challenge to the routine application of CLE.

The core aim of this study was to determine the baseline diagnostic accuracy that can be achieved with CLE alone, when assessed by neuropathologists without prior CLE training and without any additional clinical or contextual information, and to compare these findings to standard haematoxylin and eosin (H & E)‐based histology in a blinded setting. To evaluate the diagnostic accuracy and potential of CLE, we conducted this study involving neuropathologists who were not trained in CLE image interpretation and were blinded to the histological findings and all patient‐related information.

## 2 Material and Methods

### 2.1 Definition of Terms

Patient refers to an individual who underwent neurosurgical intervention for a newly diagnosed brain lesion
Case refers to a single tumour specimen obtained from a patient that was analysed using both CLE and histological H & E staining
Data set represents the complete evaluation of one case by one neuropathologist, including assessment of both CLE and H & E images. Since five neuropathologists participated in the study, each case could generate up to five data sets.

### 2.2 Study Design

Our study included 100 patients who underwent cytoreductive surgery for a newly diagnosed cerebral lesion at the University Hospital in Bern over a 22‐month period. Only patients who signed the hospital's research consent form and were aged 18 years or above at the time of the intervention were enrolled. This study, investigating CLE in an ex vivo setting (after the operation), was part of a larger study, where in vivo investigations (during the operation) were performed as well (KEK: 2018–01599).

Our primary endpoint was to determine the basic diagnostic accuracy (percentage of correct diagnoses) of CLE images without any additional information (such as patient data, tumour location, or intraoperative findings). Our true reference was the definitive histological diagnosis, based on various stains and immunohistochemical analyses.

### 2.3 CLE Image Processing

CLE imaging was performed using the CONVIVO System from Zeiss Meditec AG [18].

During surgery, tissue samples were routinely collected for standard histopathological processing. From these, one sample of approximately 0.5 cm^3^ was set aside for the study.

In a first step, the sample was placed in sodium fluorescein (FNa) (0.1%) for one minute, followed by sodium chloride (NaCl) (0.9%) for one minute. After this, CLE images were acquired by placing the sample on the fixed tip of the laser (a process referred to as “ex vivo” imaging in this study). For each sample, multiple spots at different depths were examined. The images were recorded as single images, time series, or z‐stacks. Laser power was typically set between 10% and 30%. The standard filter was a green band‐pass, and the standard gain was set at 2400.

### 2.4 Histopathology Image Processing

After CLE imaging, the tissue sample was formalin‐fixed and sent to the Institute of Pathology, where H & E‐stained slides were prepared from the same sample, following standard protocols (formalin‐fixed and paraffin‐embedded blocks).

In addition, the other samples from the same tumour were also sent to the Institute of Pathology, where the standard diagnostic workflow was carried out. This included all necessary stains, immunohistochemistry, and molecular markers required to establish the routine histological diagnosis.

### 2.5 Set‐Up of CLE and H & E Questionnaire

For each tumour, four different CLE images with minimal artefacts were selected and integrated into a “CLE questionnaire”. A second corresponding “H & E questionnaire” was created using the matching H & E‐stained images. For each questionnaire, the 100 tumours were randomly numbered and divided into four “quiz packages” of 25 tumours each to avoid any correlation between CLE and H & E images.

Both questionnaires were completed by five neuropathologists from the Institute of Neuropathology at the Charité in Berlin. All were trained in neuropathology, but had no prior experience with CLE imaging. To isolate the diagnostic accuracy of CLE, the participants were blinded to the histological findings. They also received no additional guidance (including image information, localisation, and basic patient demographics) and no feedback.

**FIGURE 1:**
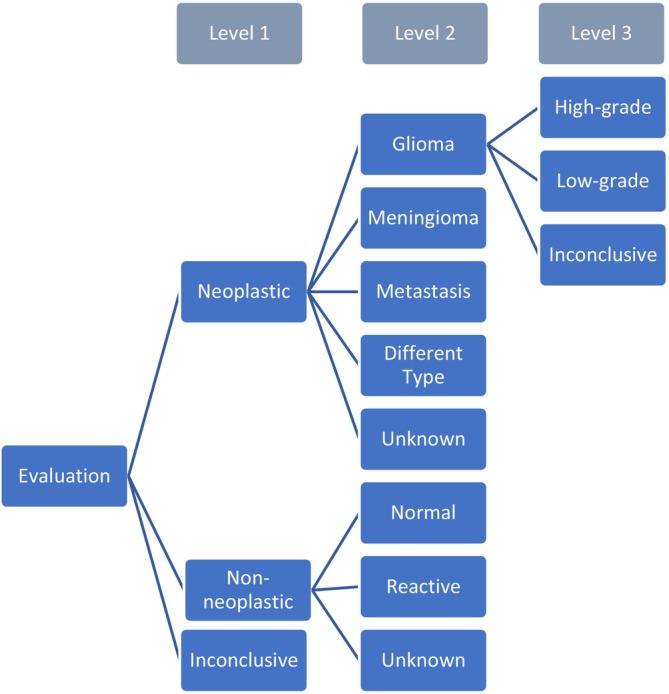
*Decision tree from questionnaire. Level 1 = malignancy, level 2 = tumour type, level 3 = grading*.

The questionnaire followed a decision tree (Figure 1), leading from malignancy (level 1) to tumour type (level 2) and grading (level 3).

Afterwards, the participants rated their confidence in their final diagnosis as “confident”, “moderately confident”, or “uncertain”. These ratings were used to calculate both overall confidence and confidence in correctly diagnosed data sets. Finally, participants could provide a presumptive diagnosis (e.g., metastasis of adenocarcinoma) and add comments or differential diagnoses.

### 2.6 Case Selection and Exclusion Criteria

To validate our cohort, we assessed the diagnostic accuracy of H & E‐stained slides, based on the assumption that if a trained neuropathologist cannot identify the tumour in H & E, they are unlikely to do so with CLE, as both show similar morphological features. Samples in which ≥ 50% of participants misdiagnosed the H & E slide were excluded from the statistical analysis, as the information was considered insufficient for reliable categorisation. Likewise, cases with tumour types other than glioma, meningioma, or metastasis were not included in the analysis, as the small sample size in this study was considered to compromise reliability.

### 2.7 Ethical Approval

This study was performed at the Department of Neurosurgery of the University Hospital Bern. Ethical approval was obtained from the local ethics committee (KEK: 2019–02291). All patients included in this study had previously given their consent for anonymised data analyses.

## 3 Results

### 3.1 Cohort Construction and Collection of Data Sets

Over a 22‐month period, 212 patients with a definitive histological diagnosis of a brain tumour underwent cytoreductive surgery at the University Hospital in Bern. Fourteen patients were excluded due to missing consent, and six patients because they were under 18 years of age, leaving 192 eligible cases.

**FIGURE 2:**
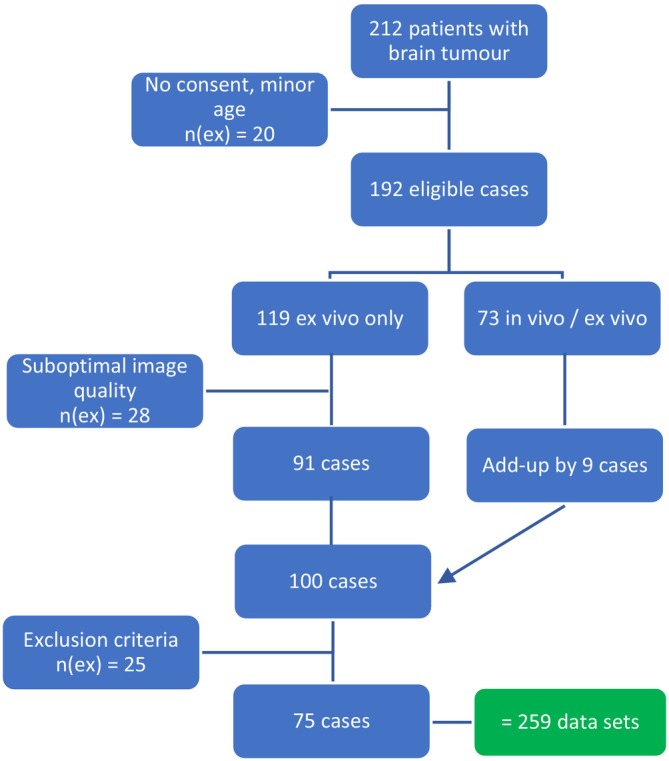
Flow process chart of cohort construction. N (ex) = number of excluded cases.

Of these, 119 were examined in an ex vivo‐only setting and 73 with both ex vivo and in vivo imaging. To reach our study cohort of 100 patients, we included all 91 ex vivo cases that met the imaging quality criteria and added the first nine cases from the ex vivo/in vivo group, which were reviewed and met the criteria. (Figure 2).

A total of 25 cases were excluded from the statistical analysis according to the predefined exclusion criteria (Table 1). This resulted in 75 patients being included in the final investigation cohort (Figure 2). The mean age of these patients (*n* = 75) was 60 years (24–91 years), and 60% were female.

**TABLE 1:** Cases removed from statistical analysis out of the patient population of 100 patients.

Patient population (*n* = 100)	No. of cases
H & E‐stained sample incorrectly diagnosed by ≥ 50% of participants	16
Other tumour types:	9
• Schwannoma	6
• Ependymoma	1
• Craniopharyngioma	1
• Chondrosarcoma	1
Total	25

In total, 259 data sets were collected. Each data set corresponded to one case evaluated by one of the five neuropathologists. With 75 cases and 5 pathologists, a theoretical maximum of 375 data sets would have been possible. Two participants (P1 and P2) each completed all 75 cases (total of 150 data sets). The remaining three participants filled out fewer cases: P3 completed 53 data sets, P4 completed 38, and P5 completed 18. Of the 259 completed data sets, 45.2% (*n* = 117) were gliomas (35.0% low‐grade gliomas, 65.0% high‐grade gliomas), 34.0% (*n* = 88) were meningiomas, 18.9% (*n* = 49) were metastases, and in five data sets (1.9%) no evidence of a tumour was found in the tissue sample.

### 3.2 Correct Answers

Participants correctly distinguished neoplastic from non‐neoplastic tissue in 70.7% (183/259 data sets). Among these neoplastic data sets, the participants identified tumour type in 47.5% (86/181 data sets). Gliomas were diagnosed correctly in 59.8% (49/82 data sets), meningiomas in 43.8% (28/64 data sets), and metastases in 25.7% (9/35 data sets). Gliomas were correctly labelled as high‐grade in 35.5% (11/31 data sets) and as low‐grade in 44.4% (8/18 data sets). Non‐neoplastic data sets were correctly diagnosed in 40.0% (2/5 data sets) (Table 2).

**TABLE 2:** Diagnostic accuracy of CLE images. Level 1 = neoplastic versus non‐neoplastic; Level 2 = tumour type (glioma vs. meningioma vs. metastasis); Level 3 = LGG versus HGG. Diagnostic accuracy of CLE images, including data sets that were marked as “inconclusive” by the pathologists on the left side, and diagnostic accuracy of CLE images excluding data sets that were marked as “inconclusive” by the pathologists on the right side(^a^).

	No. of date sets	Correctly diagnosed	Diagnostic accuracy (%)	No. of data sets^**a**^	Correctly diagnosed^a^	Diagnostic accuracy (%)^a^
Level 1:						
• Total	259	183	70.7	205	183	89.3
• Neoplastic	254	181	71.3	202	181	89.6
• Non‐neoplastic	5	2	40.0	3	2	66.7
Level 2:						
• Total	181	86	47.5	130	86	66.2
• Glioma	82	49	59.8	62	49	79.0
• Meningioma	64	28	43.8	43	28	65.1
• Metastasis	35	9	25.7	25	9	36.0
Level 3:						
• Total	49	19	38.8	35	19	54.3
• LGG	18	8	44.4	18	8	44.4
• HGG	31	11	35.5	17	11	64.7

After excluding all “inconclusive” data sets (54/259), accuracy improved: participants correctly identified the data set as neoplastic in 89.6% (181/202 data sets). Within this group, gliomas were correctly identified in 79.0% (49/62 data sets), meningiomas in 65.1% (28/43 data sets), and metastases in 36.0% (9/25 data sets). Gliomas were correctly subclassified into high‐grade in 64.7% (11/17 data sets) and low‐grade in 44.4% (8/18 data sets) (Table 2).

Sensitivity was highest for gliomas (59.8%), followed by meningiomas (43.8%). Sensitivity for metastases was 25.7%. Specificity ranged from 76.8% for gliomas to 94.9% for meningiomas. Positive and negative predictive values varied widely (Table 3).

**TABLE 3:** Sensitivity (= TP / (TP + FN)), specificity (TN / (TN + FP)), positive predictive value (PPV = TP/ (TP + FP)), and negative predictive value (NPV = TN/ (TN + FN)) of CLE images for the three most common tumour types in this study.

	Sensitivity (%)	Specificity (%)	PPV (%)	NPV (%)
Glioma	59.8	76.8	68.1	69.7
Meningioma	43.8	94.9	82.4	75.5
Metastasis	25.7	92.5	45.0	83.9

### 3.3 Incorrect Answers

Out of 73 data sets that were mistakenly not recognised as neoplastic, 28.8% (21/73 data sets) were classified as “non‐neoplastic” and 71.2% (52/73 data sets) as “inconclusive”. Among the three misdiagnosed non‐neoplastic data sets, 66.7% (2/3 data sets) were marked as “inconclusive” and 33.3% (1/3 data sets) as “glioma”.

When a neoplastic data set was misdiagnosed as “non‐neoplastic”, it was classified as “reactive tissue” in 38.1% (8/21 data sets), “normal brain tissue” in 23.8% (5/21 data sets), and in 38.1% (8/21 data sets), no further categorisation was made.

Of the 181 neoplastic data sets correctly identified at level 2 (Figure 1), the specific tumour type was misclassified in 52.5% (95/181 data sets). Among these, 34.7% were gliomas (33/95 data sets), 37.9% meningiomas (36/95 data sets), and 27.4% metastases (26/95 data sets). Of the misclassified gliomas, 15.2% were diagnosed as meningiomas (5/33 data sets), 18.2% as metastases (6/33 data sets), 6.1% as a different type (2/33 data sets), and 60.6% as unknown (20/33 data sets). Of the misclassified meningiomas, 25.0% (9/36 data sets) were believed to be gliomas, 13.9% to be metastases (5/36 data sets), 2.8% were marked as “different type” (1/36 data sets), and 58.3% were marked as “unknown” (21/36 data sets). The misclassified metastases were diagnosed as gliomas in 53.8% (14/26 data sets), as meningiomas in 3.8% (1/26 data sets), as “different type” in 3.8% (1/26 data sets), and as “unknown” in 38.5% (10/26 data sets) (Table 4). In nine data sets, the tumour type was not correctly identified at level 2 (Figure 1), while the correct diagnosis was nonetheless listed as a differential diagnosis in the handwritten comment field at the end of the case description.

**TABLE 4:** Summary of misdiagnosed data sets and corresponding pathologists' diagnoses across the three main tumour types: glioma, meningioma, and metastasis. Correct diagnosis shown on the left side, diagnosis erroneously established by the pathologists shown on the right side.

Misdiagnosed data sets	No. of data sets	Pathologists' diagnosis				
		Glioma	Meningioma	Metastasis	Different type	Unknown
Glioma	33	—	5 (15.2%)	6 (18.2%)	2 (6.1%)	20 (60.6%)
Meningioma	36	9 (25.0%)	—	5 (13.9%)	1 (2.8%)	21 (58.3%)
Metastasis	26	14 (53.8%)	1 (3.8%)	—	1 (3.8%)	10 (38.5%)

### 3.4 Validation With H & E‐Based Diagnosis

**FIGURE 3:**
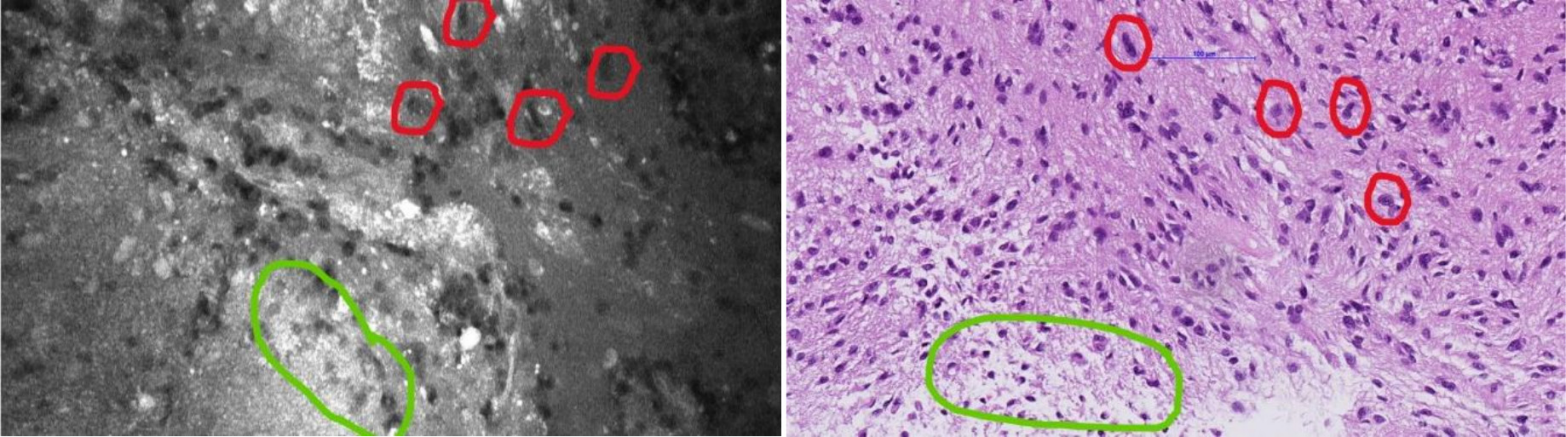
Pleomorphic cells (red) and indicated necrosis (green) in CLE image (left) and corresponding real H & E‐stained image (right). The correlation is intended to be illustrative, highlighting similar morphological features visible in both CLE and H & E images, even though the images are not taken from the exact same location or identical cells.

From the initial 100 cases, 91 remained for H & E‐based validation, as 9 cases had already been excluded due to the predefined exclusion criteria (Table 1). Two pathologists completed all 91 cases, whereas three pathologists completed 46 of the 91 cases, resulting in a total of 320 data sets. Figure 3 illustrates how the CLE and H & E cohorts correlate, underlining why H & E‐based assessment represents an appropriate validation method.

In 87.6% (276/315 data sets), the participants correctly labelled as “neoplastic”. Among these 276 data sets, the correct tumour type was identified in 89.1% (246/276 data sets). Gliomas were correctly diagnosed in 85.6% (113/132 data sets), meningiomas in 94.6% (88/93 data sets), and metastases in 88.2% (45/51 data sets). Gliomas were further correctly classified as low‐grade in 47.5% (19/40 data sets) and as high‐grade in 63.0% (46/73 data sets) (Table 5).

**TABLE 5:** Diagnostic accuracy for H & E‐stained slides. Level 1 = neoplastic versus non‐neoplastic; Level 2 = tumour type (glioma vs. meningioma vs. metastasis); Level 3 = LGG versus HGG. Diagnostic accuracy for H & E‐stained slides, including data sets where less than 50% of the pathologists diagnosed the sample correctly on the left side, and diagnostic accuracy for H & E‐stained slides excluding data sets where less than 50% of the pathologists diagnosed the sample correctly on the right side(^a^).

	No. of data sets	Correctly diagnosed	Diagnostic accuracy (%)	No. of data sets^a^	Correctly diagnosed^a^	Diagnostic accuracy (%)^a^
Level 1:						
• Total	320	279	87.2	264	255	96.6
• Neoplastic	315	276	87.6	259	252	97.3
• Non‐neoplastic	5	3	60.0	5	3	60.0
Level 2:						
• Total	276	246	89.1	252	239	94.8
• Glioma	132	113	85.6	117	108	92.3
• Meningioma	93	88	94.6	88	86	97.7
• Metastasis	51	45	88.2	47	45	95.7
Level 3:						
• Total	113	65	57.5	108	63	58.3
• LGG	40	19	47.5	36	17	47.2
• HGG	73	46	63.0	72	46	63.9

In 16 of 100 H & E‐stained specimens, ≥50% of the participants failed to identify the type correctly (level 2). Since not all five participants evaluated every case, these 16 misclassified specimens corresponded to a total of 56 data sets (16 from P1, 16 from P2, 8 from P3, 8 from P4, 8 from P5). These 56 data sets were excluded from the statistical analysis of our primary endpoint, as their inclusion would have invalidated the results (Table 5).

### 3.5 Confidence and Learning Curve

The overall confidence in diagnoses based on CLE was high in 1.5%, moderate in 29.3%, and low in 69.3% of data sets. Confidence levels were highest for metastases and lowest for gliomas. There was no significant difference between overall confidence and confidence about correctly diagnosed data sets (Table 6). The level of confidence did not increase over time, and no learning curve was observed for diagnoses based on CLE images.

**TABLE 6:** Confidence in diagnosis, subclassified into levels 1–3 (1: neoplastic vs. non‐neoplastic; 2: glioma vs. meningioma vs. metastasis; 3: LGG vs. HGG): overall confidence shown on the upper half of the table, confidence in correctly diagnosed cases shown on the lower half.

Confidence in CLE	Confident	Moderate	Uncertain	Total no.
Overall				
Level 1	3 (1.5%)	60 (29.3%)	142 (69.3%)	205
Level 2	3 (1.7%)	51 (28.2%)	127 (70.2%)	181
• Glioma	0 (0.0%)	20 (24.4%)	62 (75.6%)	82
• Meningioma	1 (1.6%)	18 (28.1%)	45 (70.3%)	64
• Metastasis	2 (5.7%)	13 (37.1%)	20 (57.1%)	35
Level 3:	0 (0.0%)	10 (20.4%)	39 (79.6%)	49
• LGG	0 (0.0%)	5 (27.8%)	13 (72.2%)	18
• HGG	0 (0.0%)	5 (16.1%)	26 (83.9%)	31
Correctly diagnosed				
Level 1	3 (1.6%)	51 (27.9%)	129 (70.5%)	183
Level 2	2 (2.3%)	19 (22.1%)	65 (75.6%)	86
• Glioma	0 (0.0%)	10 (20.4%)	39 (79.6%)	49
• Meningioma	1 (3.6%)	8 (28.6%)	19 (67.9%)	28
• Metastasis	1 (11.1%)	1 (11.1%)	7 (77.8%)	9
Level 3:	0 (0.0%)	4 (21.1%)	15 (79.0%)	19
• LGG	0 (0.0%)	3 (37.5%)	5 (62.5%)	8
• HGG	0 (0.0%)	1 (9.1%)	10 (90.9%)	11

## 4 Discussion

This initial assessment of the diagnostic value of CLE images in an observer‐blinded, reproducible ex vivo setting suggests some potential usefulness in distinguishing neoplastic from non‐neoplastic tissue and thus serving as a tool for delineating brain tumour borders. Additionally, certain CLE images with distinct histological features may assist in identifying tumour types. However, the diagnostic accuracy in CLE images was notably lower than the one achieved with H & E‐stained samples. This indicates the need for significant adjustments, such as prior CLE training and improved image quality, to enhance the reliability of CLE imaging in diagnosing brain tumours.

Our study revealed lower sensitivity and specificity of CLE‐based diagnostics compared with reports by other research groups studying the same topic [16, 17]. Unlike those studies, ours exclusively involved neuropathologists who were not trained in CLE. They also did not receive any additional information, such as patient age, MRI contrast enhancement, or lesion localisation. These factors, along with our broad patient group, likely contributed to the observed discrepancies. Furthermore, our neuropathologists did not receive any feedback during the study, resulting in low overall confidence in diagnoses based on CLE images and the absence of a discernible learning curve. Given the complexity of CLE images, prior training in their interpretation is highly recommended. With increasing experience and specific CLE training, a notable improvement in diagnostic accuracy and confidence levels might be anticipated.

By blinding the neuropathologists, this study aimed to independently assess baseline diagnostic accuracy. We deliberately chose to investigate the core question: what level of diagnostic accuracy can CLE achieve when all additional clinical information is removed? While neuropathologists can usually interpret H & E slides with high accuracy even without further context, in this study, CLE did not reach a comparable level. However, this approach does not replicate real‐world conditions, where pathologists routinely familiarise themselves with tumour location, imaging findings, and basic patient history to achieve the best possible intraoperative outcome. The blinding throughout the study process, therefore distances the study setting from clinical practice. This can also be seen in the error rate of the H & E validation.

In this study, we evaluated CLE images, which were generated using samples after topical application of FNa for staining. In the above‐mentioned studies, staining was performed by intravenous injection of FNa. While intravenous application highlights vascular and extracellular detail due to systemic distribution, topical application enhances intracellular and nuclear structures. This difference in protocol may affect image contrast and resolution, possibly leading to different diagnostic conclusions [19]. In summary, the specific features highlighted by topical staining are not directly comparable to those revealed by intravenous staining, which better reflects the “real‐life scenario”.

In our study, we deliberately conducted our research in an ex vivo environment to minimise artefacts in the images arising from motion, time constraints, and blood cell contamination. Still, a recently published phase II trial performed CLE in an in vivo setting with the application of intravenous FNa and reported a diagnostic accuracy of 87% in intraoperative histological diagnosis. [20]

The primary purpose of CLE is to distinguish between neoplastic and non‐neoplastic tissue and to support the surgeon in achieving the most complete resection possible. However, when CLE is used to identify specific tumour types or reproduce detailed histological features, it is important to keep in mind the recent changes introduced by the 5th WHO classification [21]. According to this, histological morphology alone is no longer sufficient for brain tumour classification, as molecular markers and methylation‐based profiling now play a central role [22].

It is important to note that, in current clinical practice, tumour resections are planned and guided using advanced imaging modalities such as contrast‐enhanced MRI and 5‐ALA fluorescence. In this context, future studies may be needed to explore how CLE could complement these established techniques and whether its findings can be meaningfully correlated with intraoperative imaging guidance.

## 5 Conclusion

CLE imaging demonstrates promise in differentiating between neoplastic and non‐neoplastic tissue, although with significantly lower accuracy than that achieved with the corresponding H & E‐stained slides. To improve diagnostic accuracy, prior training in CLE image interpretation is recommended. Additionally, it should be noted that at the moment, the use of FNa remains off‐label, and reimbursement issues related to CLE use in neurosurgical diagnostics have yet to be resolved. Currently, further refinements in CLE technology to achieve an in vivo image quality comparable to an ex vivo setting are needed and are underway. Based on our results, we conclude that there is potential for CLE imaging to emerge as a valuable tool for delineating brain tumour borders.

## Author Contributions

**Ilona Iff:** Data collection, data evaluation, manuscript writing. **Marielena Gutt‐Will:** Study design, data evaluation, manuscript writing. **Theoni Maragkou:** Data collection, data evaluation. **Andrea Mathis:** Data collection, data evaluation, manuscript writing. **Kathleen Seidel:** Data collection. **David Capper:** Data collection. **Elisabeth G. Hain:** Data collection. **Jenny Meinhardt:** Data collection. **Regina Von Manitius:** Data collection. **Carsten Dittmayer:** Data collection. **Simone Schmid:** Data collection. **Ekkehard Hewer:** Data evaluation. **Andreas Raabe:** Study design, overall responsibility. **Philippe Schucht:** Study design, data collection, manuscript writing, overall responsibility.

## Funding

This study was part of an overall project which was financially supported with 40′000 CHF per year, as well as by providing the CLE equipment by Carl Zeiss Meditec AG.

## Conflicts of Interest

The authors declare no conflicts of interest.

## Data Availability

The data that support the findings of this study are available from the corresponding author upon reasonable request.
